# Collagen XV, a multifaceted multiplexin present across tissues and species

**DOI:** 10.1016/j.mbplus.2020.100023

**Published:** 2020-01-15

**Authors:** Sandrine Bretaud, Emilie Guillon, Sanna-Maria Karppinen, Taina Pihlajaniemi, Florence Ruggiero

**Affiliations:** a Institut de Génomique Fonctionnelle de Lyon, ENS de Lyon, UMR CNRS 5242, University of Lyon, Lyon 69364, France; b Center for Cell-Matrix Research, Faculty of Biochemistry and Molecular Medicine, University of Oulu, Aapistie 7C, FI-90230 Oulu, Finland

**Keywords:** Collagens, Multiplexin, Extracellular matrix, Animal models, Development, Collagen-related disease, Evolution, BM, basement membrane, BMZ, basement membrane zone, CS, chondroitin sulfate, CSPG, chondroitin sulfate proteoglycan, dpf, day post-fertilization, ECM, extracellular matrix, GAG, glycosaminoglycan, HFD, High fat diet, HS, heparan sulfate, HSPG, heparan sulfate proteoglycan, COL, collagenous domain, NC, non-collagenous domain, TD, trimerization domain, TSPN, Thrombospondin-1 N-terminal like domain

## Abstract

Type XV collagen is a non-fibrillar collagen that is associated with basement membranes and belongs to the multiplexin subset of the collagen superfamily. Collagen XV was initially studied because of its sequence homology with collagen XVIII/endostatin whose anti-angiogenic and anti-tumorigenic properties were subjects of wide interest in the past years. But during the last fifteen years, collagen XV has gained growing attention with increasing number of studies that have attributed new functions to this widely distributed collagen/proteoglycan hybrid molecule. Despite the cumulative evidence of its functional pleiotropy and its evolutionary conserved function, no review compiling the current state of the art about collagen XV is currently available. Here, we thus provide the first comprehensive view of the knowledge gathered so far on the molecular structure, tissue distribution and functions of collagen XV in development, tissue homeostasis and disease with an evolutionary perspective. We hope that our review will open new roads for promising research on collagen XV in the coming years.

## Highlights

• Type XV collagen belongs to the multiplexin subset of the collagen superfamily.
• It is evolutionarily conserved collagen and associated with basement membranes.
• This collagen/proteoglycan hybrid molecule contains an anti-angiogenic restin domain.
• It has important functions in the cardiovascular and the neuromuscular systems.
• Its expression is dysregulated in various diseases including cancers.

## Introduction

When the public, research communities or even biologists not specialized in the collagen field think about collagen, they mostly unknowingly refer to fibrillar collagens. This subset of the collagen superfamily clearly represents essential components of almost every extracellular matrix environment and as such they have been extensively studied over decades. Countless number of reviews have discussed their structure and functions including recent state-of-the-art reviews [1,2]. However, the collagen superfamily comprises not less than 28 collagen types that display various molecular structures, network organization and tissue distribution. The non-fibrillar collagens are way less understood, but individually gain more and more interest for their diversity of physiological and pathological functions in development, tissue homeostasis, repair and disease. Type XV collagen belongs to the subgroup of evolutionary conserved non-fibrillar basement membrane (BM) associated collagens, known as multiplexins (multiple triple-helix domains with interruptions), together with the homologous collagen XVIII [3,4]. The zebrafish genome contains several paralogs for the human collagen XV and XVIII genes [5,6] while only one multiplexin exists in invertebrates [7,8]. Despite these differences, the expression pattern and function of collagen XV showed considerable similarities across species [[7], [8], [9], [10], [11], [12]].

Collagen XV has a complex multidomain structure that shows specific structural features [3,4]. First, it is a proteoglycan/collagen hybrid molecule that contains mainly chondroitin sulfate chains [13]. Second, the C-terminal region of collagen XV contains a trimerization domain and a restin domain, which is homologous to the endostatin fragment of collagen XVIII that possesses anti-angiogenic and anti-tumorigenic properties [14]. Third, the molecule contains multiple triple-helix domains interrupted by non-collagenous sequences.

Collagen XV has been shown to play an important structural role in maintaining the integrity of the extracellular matrix (ECM), but has also significance in a number of physiological and pathological processes. Various vertebrate and invertebrate animal models have been used to interrogate the function of collagen XV in development and disease. Knockout mice for *Col15a1*^*−/−*^ showed mild skeletal myopathy and cardiovascular defects. Specifically, the micro-vessels of skeletal muscles and heart of these mice presented several abnormalities, including endothelial cell degeneration, increased vessel permeability and collapsed capillaries [15,16]. Furthermore, lack of collagen XV in mice affected nerve maturation and provoked abnormal myelination [17]. Remarkably, studies using other animal models have revealed that collagen XV preserves its function in the cardiovascular and neuromuscular systems throughout evolution [7,8,12,18]. In addition, several studies have suggested that collagen XV acts as a tumor suppressor [19], and a recent study showed a role for it in the development of atherosclerotic lesions [20]. The gene encoding collagen XV was identified as a modifier of the severity of thoracic aortic aneurysms [102] and as a candidate gene for age-related macular degeneration [89]. However, to date, there is no human disorder associated with mutations in the *COL15A1* gene. Overall, these data provide increased understanding of the multiple functions of collagen XV both in health and disease and point towards possible new disease associations for collagen XV.

Here, we summarize the gene and protein structure of collagen XV across species and review the current knowledge of its function with an evolutionary aspect. Moreover, the roles of collagen XV in the context of diseases and pathological conditions, with largely unknown molecular background, will be discussed.

## The multiplexin collagen XV: structural features and conformational characteristics

### From gene to quaternary structure in different species

**Fig. 1:**
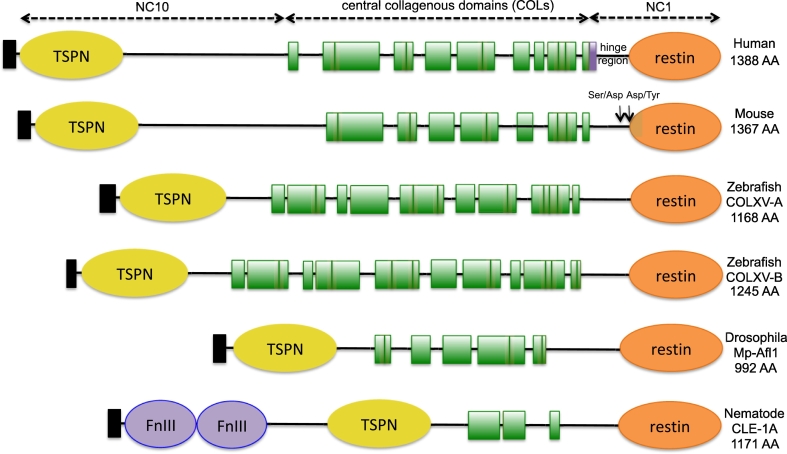
Schematic representation of the structure of collagen XV chains across species. Peptide signals are represented by black rectangles. Collagenous domains (COL) are represented in green. Interruption of COL domains corresponds to linker regions. Short interruptions (1 to 2 triplets) within the triple helical domains are indicated with green vertical lines. The N and C-terminal non-collagenous (NC) domains that form distinct globular domains are highlighted in yellow for thrombospondin-1 N-terminal like (TSPN), orange for restin and purple for fibronectin type III like (FnIII) domains. The trimerization domain (purple rectangle) was characterized for the human protein [27]. Arrows indicate the potential proteolytic cleavage sites of restin identified in the mouse recombinant protein (arrows) [31]. AA, amino-acid.

Collagen XV was first described in mice and humans. It is a homotrimer consisting of three α1 polypeptide chains [α1(XV)]_3_ encoded by the *COL15A1/Col15a1* gene localized respectively in the *9q21-q22* chromosomic region in humans and the *4B1-B3* region in mice [[21], [22], [23], [24], [25]]. *COL15A1* is a 145-kilobase gene containing 42 exons coding for one polypeptide chain of 1388 amino acids. The human α1(XV) chain consists of nine triple helical regions, termed COL domains that are numbered starting from the C-terminus (COL1-COL9). These COL domains that are characterized by the repetition of the (Gly-X-Y) triplet sequence alternate with non-collagenous (NC) domains. NC domains are short sequences varying in size (from 7 to 45 residues) that are considered as linker regions (Fig. 1) [24,25]. The murine α1(XV) chain, made of 1367 amino acids, slightly differs from the human chain as it contains only seven COL domains (Fig. 1), but the overall α1(XV) chain sequences are highly conserved between the two species, with sequence similarity reaching 91% [22]. Additionally, COL domains contain interruptions of about 2–3 residues in the repeating (Gly-X-Y) sequence (Fig. 1). These interruptions cause local disruptions that may increase sensitivity of collagen XV to proteases. The central COL region is flanked by two larger NC domains that comprise subdomains: the NC10 and NC1 domains at the N-terminus and C-terminus, respectively (Fig. 1). NC10 comprises the globular TSPN domain that is homologous to the N-terminal domain of thrombospondin-1 and is present in several other collagen types [26]. The functional significance of this domain remains still unclear.

The NC1 region contains a trimerization domain (TD), a hinge region and a restin domain (Fig. 1). The TD is critical to initiate in-register (*i.e.* aligned strands) triple helix assembly [27]. The sequence of the human collagen XV TD is however much shorter (around 50 residues) [27] than the ones of the fibril-forming collagens ranging in size from 160 to 230 residues [28,29]. The crystal structures of the multiplexin TDs (collagen XV and collagen XVIII) show a similar 3D structure despite the fact that they share only 32% primary sequence homology. They both contain an atypical hydrophobic core that is involved in the stabilization of the oligomeric structure [27]. This structural property has been used as a strategy for advanced engineering to produce collagen XV trimeric antibodies (trimerbodies) which are expected to exhibit improved pharmacokinetic properties [30]. The C-terminal part of the NC1 domain can be cleaved proteolytically in a hinge region resulting in a biologically active fragment known as restin (described in details hereafter), which is homologous to the collagenXVIII/endostatin domain [14,31]. In addition, collagen XV is characterized in all species by the presence of highly conserved cysteine residues localized within the TSPN and NC1 domains that are likely responsible for intra-chain disulfide bonds as shown for mammalian proteins. However, mammalian collagen XV also contains two additional cysteine residues within the central COL region that form interchain disulfide bonds [13].

The multiplexins are conserved across species. In zebrafish, representing a lower vertebrate, two paralogs of *COL15A1*, *col15a1a* and *col15a1b*, encode the COLXV-A and COLXV-B proteins respectively [9,10]. Using clustal omega, we showed that the two proteins share respectively 44 and 50% identity with the human COLXV. The overall structure of the zebrafish paralogs is well conserved although the analysis of the COLXV-B predicted from the *col15a1b* gene (accession number LK391962, [18]) revealed the presence of an additional COL domain (Fig. 1). In invertebrate animal models, *Caenorhabditis elegans* and *Drosophila melanogaster*, there is only one gene orthologous to the two human multiplexin genes, *COL15A1* and *COL18A1*, named *cle-1* and *dmp/mp* respectively [7,8,11]. The central COL domain of the collagen XV/XVIII orthologs contains only 5 COL domains for Mp-Afl1 in *Drosophila* and 3 COL domains for CLE-1A in *C. elegans* (Fig. 1). Multiple transcripts encoding several isoforms that highly differ from the mammalian protein exist *in vivo* including a Mp isoform in which the central COL domain is absent and a CLE isoform containing additional fibronectin type III (FNIII) repeats [7,11].

Rotary shadowing electron microscopy has been used to analyze the native human type XV collagen extracted from umbilical cord [32] or the human protein produced recombinantly in insect cells [33]. Collagen XV molecule is particularly flexible due to the presence of numerous COL domain interruptions (Fig. 1). Rotary shadowing electron microscopy revealed that it displays a pretzel-like shape that has never been described for other collagen types [32,33]. These experiments have also revealed that collagen XV molecules assemble into compact multimers with a cruciform appearance resulting from association of two to four molecules. Interestingly, the TSPN domains often stick out from the multimeric structures, favouring their interactions with potential molecular and cellular partners, though no evidence for such interactions is available so far.

### The restin domain: a matrix matricryptin with specific features

A fragment of 185 residues of the C-terminal part of human collagen XV NC1 was shown to share 60% identity with endostatin and was thus named restin for “related to endostatin” [34]. The restin domain is also the most conserved collagen XV sequence across species; for instance the human restin is 61% identical with the one of the zebrafish COLXV-B protein [10]. Both restin and endostatin are matricryptins. This term refers specifically to biologically active extracellular matrix fragments that result from proteolytic cleavage of the parental ECM proteins [35]. While endostatin can be easily released from collagen XVIII-NC1 because of the high sensitivity to protease of the hinge region connecting the matricryptin, this is not the case for collagen XV. Indeed, the hinge region is much shorter (~20 residues) with only two potential proteolysis sites identified for COLXV against 11 for COLXVIII [31,36]. Such a difference correlated with the substantial presence of endostatin in tissues contrarily to the absence of restin except in human plasma [37].

Both recombinant mouse NC1 and restin domains were produced to determine their 3D structure using X-ray crystallography [31]. Restin folds as a globular structure of 2 nm diameter similar to the endostatin domain. However, some differences were observed, for example restin lacks the zinc and heparin binding sites of endostatin (even if some of the arginin residues important for heparin binding are conserved between endostatin and restin sequences). Two phenylalanine residues present at the surface of the domain are conserved in both restin and endostatin and were suggested to correspond to a putative receptor binding site [31]. Indeed, the two phenylalanines of endostatin were later shown to represent the androgen receptor recognition motif called NR box [38]. It is interesting to note that the analysis of the restin domain of all animal models shows a well-conserved NR box with the presence of an aromatic tyrosine residue in some cases instead of phenylalanine (unpublished data). Type XV and XVIII collagens are both components of BMs. The binding of the NC1 and restin domains to BM proteins, such as laminin 1, nidogens, fibulins and perlecan, has thus been investigated in details and compared to their collagen XVIII counterparts. The NC1 domain of collagen XV bound to almost all the components tested at comparable levels to restin except for fibulin 1 for which restin shows an eightfold higher binding. Both domains, NC1 and restin, showed particular strong binding to nidogen 2 and fibulin 2 [31]. The NC1 domain of collagen XVIII also bound to all BM components but, contrary to NC1-XV domain, showed very strong binding to perlecan, nidogen 1 and laminin 1. Endostatin interacted weakly with all these components except fibulins [31]. These interactions are essential for the structural and functional integrity of BMs. The release of the restin and/or endostatin from the parental molecules can occur in a variety of different biological processes and, as such, can result in significant alteration of BM integrity and function.

### Collagen XV, a collagen/proteoglycan hybrid

Human collagen XV carries glycosaminoglycan chains (GAGs), just like true proteoglycans [13,39]. Analysis of the human collagen XV using rotary shadowing electron microscopy has confirmed the presence of GAGs at the surface of both collagen XV monomers and aggregates [32]. Their relative accessibility at the surface of the multimers suggests that they may substantially contribute to the function of collagen XV *in vivo*. GAGs are involved in the regulation of numerous biological processes, the most thoroughly described of which are axon growth and angiogenesis [40,41]. They contribute to the regulation of signaling pathways (such as the FGFs, Hedgehog, or Wnt/ß-catenin pathways) as, strictly speaking, signaling molecules, as co-receptors, or else as regulators of the availability of soluble factors [42,43]. Moreover, since these chains have a high hydration coefficient, they contribute to the ECM structural organization by modifying biomechanical properties and/or porosity of matrix networks [44]. Individual collagen XV functions can thus be attributed to either its protein core or its attached GAG chains.

*In silico* analyses have indicated eighteen putative sites of glycosaminoglycans attachment to the human α1(XV) chain (including eight sites containing the consensus D/*E*-X1-2-S-G/A sequence), mainly in the N-terminal region [13,32]. Human collagen XV, which presents as a clear band at 250 kDa after digestion with chondroitinase, initially migrates in the form of a smear at about 450 kDa. It has been thus estimated that each human α1(XV) chain carries 4 to 20 GAG chains [13]. In contrast, even though the murine α1(XV) chain contains four sequences which may correspond to GAG attachment consensus sites, there are no experimental data indicating that the murine collagen XV is indeed a chondroitin sulfate proteoglycan (CSPG). Human collagen XV can also contain a combination of chondroitin sulfate (CS) and heparan sulfate (HS) chains making it a chondroitin sulfate-heparan sulfate hybrid proteoglycan, the CS/HS ratios varying between different tissue types [13,32,39]. For example CS chains are the majority in the umbilical cord, placenta and colon, while HS chains dominate in the kidney [32,39].

Collagen XVIII is exclusively a heparan sulfate proteoglycan (HSPG) [45]. This difference may have major consequences for the functions of these two collagen types given that the biological effects of the two molecules in question are sometimes opposite or complementary [46]. Interestingly, the proteoglycan hybrid properties of multiplexins seem to have been conserved during evolution. Indeed, the *Drosophila* multiplexin, Dmp/Mp, does contain consensus GAG attachment sites and biochemical analysis of the protein extracted from embryonic tissues revealed the presence of CS chains only, making it more similar to human collagen XV than collagen XVIII in this respect [11]. In zebrafish, the recombinant protein COLXV-B appeared as a high molecular weight smear typical for proteoglycan [18].

## A multiplexin with a widespread tissue distribution across species

Numerous studies have described the expression of collagen XV in human and murine tissues during development and in adults, analyzing collagen XV expression both at the transcript and protein levels. Collagen XV is expressed in nearly all studied organs. This wide expression pattern seems to be quite well conserved between mice and humans, even though comparisons cannot be made for all organs due to incomplete data. A summary of the key data obtained in these studies is presented in Table 1. These data represent important clues for further interpreting phenotypes of genetically-modified animals and to interrogate the multiple functions of this protein.

**Table 1:** Distribution of collagen type XV in mammalian tissues and organs. NB, Northern blot; IF, Immunofluorescence; IHC, immunohistochemistry; IG, immunogold electron microscopy; ISH, *in situ* hybridization; PNS, peripheral nervous system.

Organ/tissue	Human		Mouse	
	Developing	Adult	Developing	Adult
Brain	NB [47]			
PNS			IF [50]	IF [50]
Cornea		IHC [76,99] IF [63]	IF [100]	
Heart	ISH, IF [49] NB [47]	NB [47,48]	IF [50]	NB [22] IF [50]
Skeletal muscle		ISH, IF [49] NB [47,48]	IF [50]	NB [22] IF [50]
Lung	ISH, IF [49] NB [47]	NB [48] IF [51]	IF [50]	NB [22]
Pancreas	ISH, IF [49] NB [101]	NB [47,48]		
Intestine		IG [39] NB [48] IF [51]		
Adrenal gland	NB [101]			
Kidney	ISH, IF [49] NB [47,101]	IG [39] NB [47,48] IF [51]	IF [50]	NB [22] IF [50]
Placenta		IG [39] ISH [49] NB [47,48] IF [49,51]		
Skin		IHC [79] ISH, IF, [49,51]		
Ovary		NB [48]		
Testis		NB [48]		NB [22]
Prostate		NB [48]		
Cartilage and Bones		IHC [53]	IF [50]	

### Collagen XV is a basement membrane-associated collagen

In human, collagen XV is expressed by a wide variety of cell types: connective tissue cells, such as fibroblasts, cardiac, skeletal and smooth muscle cells, osteoblasts and adipocytes but also epithelial cells, endothelial cells and neuronal cells are all capable of producing collagen XV [47]. Collagen XV primary localizes to peripheral nerves in mouse and is highly expressed in cardiac and skeletal muscles both in human and mouse [[48], [49], [50]] in adults and/or during the development, which suggests that it plays a decisive role in the formation and maintenance of these tissues/organs. It is mainly associated with basement membrane zones (BMZ), although it can also be found associated with fibrillar collagen in some tissues. Immunogold labeling of human kidney, colon, and placenta tissues has revealed that collagen XV is almost exclusively associated with the fibrillar collagen network in the connective tissue in close proximity to the outer surface of the BM [39]. It has been proposed that collagen XV serves as a structural link between the cells that produce it and the underlying connective tissue. The authors also suggested that, due to the presence of the CS and HS chains, collagen XV may contribute to maintaining the porous network underlying the BM, that is essential for the diffusion of extracellular signaling molecules [39].

Because of the high homology between the two multiplexins, collagen XV and XVIII, monoclonal antibodies were raised to analyze their tissue-specific distribution and overlaps [51]. While type XV collagen mainly localized in skeletal and cardiac muscles, type XVIII was observed in subepithelial BMZ (kidney, placenta, lung, skin, liver). Specialized capillaries of the kidney, liver, lung and spleen did not contain any collagen XV. The observed differences in the tissue distribution further support the distinct functions observed for the two multiplexins although they are structurally closely related [46].

### Localization of collagen XV in the neuromuscular system

Immunofluorescence studies showed that collagen XV is highly present in the BMZ surrounding each muscle fiber of both mouse and human skeletal muscles [50,51]. In humans, *COL15A1* transcripts were detected in mononuclear myocytes, multinucleated muscle fibers and fibroblasts [47]. In mice, the collagen XV deposition started with the formation of myotubes in the embryo and increased progressively during the development. The signal persisted in the BMZ surrounding the myofibers in adults [50].

In mice, collagen XV was also present in the peripheral nervous structures. It was found in connective tissues surrounding the ganglia during embryonic development and in those surrounding adult intramuscular peripheral nerves (endoneurium and perineurium). The concomitant localization of collagen XV at the BMs of the muscles and of the nerves suggested that collagen XV may be present at the interface between muscle and motor nerve, at the neuromuscular junction. However, co-staining of collagen XV and acetylcholine receptors present at the neuromuscular junction showed that collagen XV is absent from the synaptic BMZ and is rather confined to the extrasynaptic BMZ and to those surrounding Schwann cells [50].

Of the two multiplexins, the expression in muscles is specific to collagen XV. Collagen XVIII is associated with BMZ of epithelial and endothelial cells and it has never been described as a component of any type of muscle tissues [51].

### Collagen XV, a marker of osteogenic differentiation

In mouse, collagen XV was originally shown to be present in the perichondrium of the cartilage primordial of long and short bones that undergo endochondral ossification [50]. In an attempt to identify new markers of bone marrow mesenchymal stem cells, *COL15A1* was shown to be expressed in stem cells when they are already engaged in osteogenic differentiation. This multiplexin was even the most up-regulated gene in osteoblasts [52,53]. Bone tissue biopsies confirmed the *in vivo* presence of collagen XV in osteoblasts [53]. Immunogold localization of collagen XV during *in vitro* osteogenic differentiation revealed collagen XV in association with fibrillar components of ECM, far from mineralized nodules [54]. Collagen XV expression in cultured human osteoblasts was down-modulated by increasing concentrations of extracellular calcium suggesting a possible negative correlation between collagen XV expression level and tissue calcification [55,56]. However, the mechanism by which extracellular calcium regulates collagen XV expression has not been elucidated yet. In the early-phases of the osteogenic process, collagen XV may participate in ECM organization that represents a prerequisite to the subsequent bone mineralization [56].

### Collagen XV distribution in lower species

In zebrafish, the two paralogs *col15a1a* and *col15a1b* showed distinct expression patterns [9,10]. Whereas *col15a1a* transcripts were restricted to the notochord during early embryogenesis [9], *col15a1b* was expressed in various organs during embryonic development such as skeletal muscle, heart, brain, eyes, otic placodes and aortic arches [10]. Specifically, the second paralog *col15a1b* was transiently expressed by slow muscle progenitors, called adaxial cells because of their location adjacent to the notochord. Upon Shh signal from the notochord, these cells underwent a series of stereotyped cell rearrangements before they migrated through the lateral somite to reach the periphery of the myotome where they differentiate into slow muscle and have ceased to express *col15a1b*. Interestingly, Guillon and collaborators showed that the protein COLXV-B is then deposited in a polarized way into the motor path, an ECM specialized region where growing motor axons exit from the spinal cord and extend along a stereotyped trajectory to innervate the myotome. The authors demonstrated that *col15a1b* expression and the mechanism directing protein deposition in the common motor path are dependent on a novel two-step mechanism involving Hedgehog/Gli and unplugged/MuSK signaling pathways, respectively [18]. Zebrafish and humans use common molecular cues and regulatory mechanisms for the neuromuscular system development and mammalian collagen XV was also shown to be present along peripheral nerves. However, the exclusive distribution of collagen XV along zebrafish motor nerve and not surrounding muscle cells as in mammals may reflect evolutive changes.

**Fig. 2:**
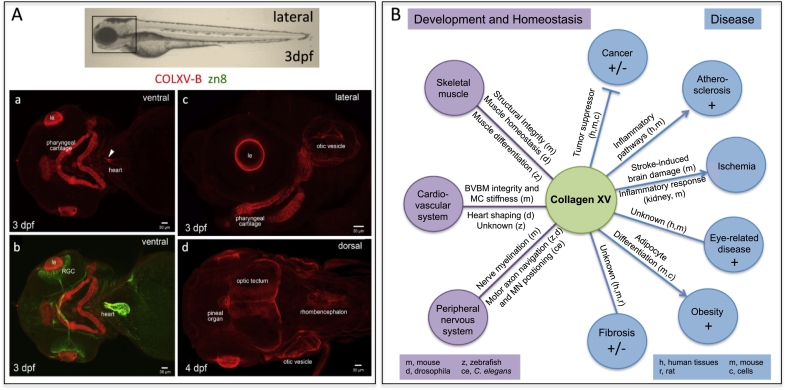
Tissue distribution and function of collagen XV. A. Collagen XV-B localization in the nervous system and other tissues of zebrafish larvae (a, b, c: 3dpf; d, 4dpf) after immunostaining with anti-COLXV-B (red; a–d) and zn8 antibody (green; b) as described [18]. Upper panel, Bright field image of a 3dpf larva, box indicates the region observed in a–d. a, Arrowhead indicates the presence of COLXV-B in the bulbous arteriosus of the heart and its connection to the ventricle (ventral view). b, Merge signal with zn8 antibody (green) used to label the retinal ganglion cells (RGC) and heart. c, COLXV-B deposition around the lens (le), in the otic vesicle and in the pharyngeal cartilage (lateral view). d, COLXV-B is present in the central nervous system especially covering the pineal organ, in the peripheral midbrain layer of the optic tectum and in the rhombencephalon (dorsal view). Anterior is on the left side. B. Pleiotropic and evolutionary conserved functions of collagen XV in development homeostasis and disease. BVBM, blood vessel basement membrane; MC, myocardium; MN, motoneuron; +, upregulated expression; +/−, tissue-dependent dysregulated expression.

Whereas brain structures in mammals were only positive during development, COLXV-B deposition was observed in sensorial organs (eye and otic vesicles) at post-embryonic stages, in larvae (Fig. 2A) confirming *in situ* hybridization results [10]. Moreover, the pineal organ and the optic tectum were clearly surrounded by COLXV-B deposits (Fig. 2A). Of note, zebrafish COLXV-B was also present in a restricted area of the heart corresponding to the bulbous arteriosus and its connection to the ventricle (Fig. 2A).

In invertebrates, the multiplexin Dmp/Mp in flies [8,11,12] and CLE-1 in worms [7] are also widely distributed in BMZ. The multiplexin Dmp/Mp was present in the heart, visceral muscle and vessels while the *C. elegans* multiplexin was described in the pharynx, body wall muscle and intestine. Strikingly, the invertebrate multiplexins were reported in the central and peripheral nervous system supporting a conserved neuronal tissue distribution across evolution.

### Collagen XV is expressed by stem cells and progenitors

There is growing evidence that ECM contributes to the differentiation and maintenance of stem cells [57]. The contribution of human and mouse collagen XV to stem cell and cancer stem cell function has been recently reviewed [58], and is mainly reported in cells of mesenchymal origin. As discussed above, *col15a1b* is highly expressed by slow muscle progenitors, the so-called adaxial cells, and is part of the slow muscle differentiation program [10,18]. Zebrafish has also become an interesting model to interrogate the function of collagen XV in neural stem cells. Zebrafish *col15a1b* was originally identified as *mz98*, a marker of the ciliary marginal zone (CMZ) localized at the periphery of the retina [59]. *In situ* hybridizations confirmed *col15a1b* expression in CMZ and in the optic tectum [10] and COLXV-B immunoreactivity was detected in the optic tectum and surrounding the lens (Fig. 2A). The CMZ and the optic tectum share common molecular signatures and express numerous canonical proliferation markers [60]. Interestingly, CMZ maintenance was recently shown to be controlled by the level of hypoxia in zebrafish embryos [61]. Similarly, collagen XV expression was directly controlled by HIF-1α (hypoxia-inducible factor-1 alpha) in response to hypoxia in human preconditioned-mesenchymal stem cells [62]. It is thus tempting to postulate that COLXV-B represents a key component of the stem cell niche. In support to this assumption, collagen XV is also expressed in human corneal limbal epithelial cells and amniotic membrane epithelium that both represent a source of stem cells and progenitors [63].

## What functions for collagen XV?

To understand the function of collagen XV, various genetically-modified animals have been created. The generation of a *Col15a1* knock-out mouse line was first reported in 2001 [15] and mutants were further obtained in *Drosophila* and *C. elegans* [7,8,11], and more recently in zebrafish [18]. The use of diverse animal models, invertebrates and vertebrates, has proven to be useful to fully understand the complexity of the *in vivo* functions of collagen XV and to reveal its conserved functions (Fig. 2B). As already mentioned, although the two vertebrate multiplexins are structurally closely related, it has been shown that double knockout of *Col15a1* and *Col18a1* genes in mice revealed a lack of major functional compensation [46] but rather complementary functions between them.

### Skeletal muscles

*Col15a1* knock-out in mice results in mild myopathy [15]. From the histological point of view, this myopathy is characterized by a mild muscular atrophy, degeneration of muscle fibers, macrophage infiltration, and the presence of muscle fibers with centrally located nucleus, the sign of skeletal muscle regeneration. These alterations occur in two-thirds of the mutant mice, from the age of three months, and intensify with age. The mice move normally but the muscular damage increases substantially when the mice are submitted to intensive exercise, which suggests the structural role of collagen XV. The attachment of muscular fibers to the surrounding ECM might become more fragile in the absence of collagen XV and cause muscle degeneration observed in the mutant mice. However, immunostaining of different BM components showed that lack of collagen XV does not impair the organization and structure of the muscle fiber BM. Moreover, the BM around degenerating fibers remains intact, which indicates that the muscle fiber fragility zone is rather likely to be localized between the BM and the connective tissue. This agrees with its location at the outer surface of BMs. Collagen XV could thus ensure a structural continuity between the BM and collagen fibers of the connective tissue.

Furthermore, the capillaries irrigating striated skeletal muscles in the *Col15a1*^*−/−*^ mice display defects such as collapse and endothelial cell degeneration [15]. These alterations affect the vascular permeability and blood microcirculation in striated skeletal muscles [64]. Therefore, one may hypothesize that the mild myopathy observed in the *Col15a1*^*−/−*^ mice could be partly related to the perfusion defect in the striated skeletal muscles.

Studies in *Drosophila* opened new interesting perspectives regarding the contribution of collagen XV to the regulation of signaling pathways involved in the maintenance of skeletal muscle homeostasis. Drosophila Mp mutants, like the *Col15a1*^*−/−*^ mice, showed progressive deterioration of muscular function and myopathy [65]. However, such a phenotype could be a consequence of mitochondrial degeneration caused by the impairment of the ßPS integrin (the orthologue of the ß-integrin in vertebrates) signaling pathway and enhanced production of free radicals that was also observed in the muscle fibers [65]. Nevertheless, it cannot be excluded that these intracellular disturbances do not reflect the signaling function of Dmp/Mp but are rather indirect consequences of a defective anchorage of muscle fibers in the surrounding connective tissue. In zebrafish, the function of the COLXV-A paralog in skeletal muscle is conserved, even if collagen XV is not expressed in developing muscle cells like in mammals. Instead, the zebrafish COLXV-A was deposited at the BM of the developing notochord next to the myotome and indirectly influenced muscle development by interplaying with Shh signaling emanating from the notochord [9].

### Cardiovascular system

Detailed analyses of the *Col15a1*^*−/−*^ mouse phenotype [15] revealed that the mutant mice also suffered from a mild but complex cardiomyopathy showing progressive symptoms and signs [16]. This is not surprising considering that collagen XV is a component of the cardiomyocyte BM. Moreover, the mutant mice are predisposed to cardiac tissue degeneration under cardiac stress, such as vigorous exercise. The cardiomyopathy developed by the *Col15a1*^*−/−*^ mice is essentially characterized by structural alterations of the myocardium and cardiac capillaries. The latter showed a decrease in capillary diameter and endothelial cell morphological abnormalities (cell swelling/folding) or degeneration. Based on these observations, it has been hypothesized that collagen XV helps to maintain the BM integrity of cardiac capillary endothelial cells. These structural defects have hemodynamic consequences giving rise to local perfusion impairments and leading to ischemic damage of cardiomyocytes. In addition to the damages indirectly caused by the lack of proper perfusion of the cardiomyocytes, the myocardium suffers from structural alterations both at the cell and tissue levels. At the cell level, lack of collagen XV disrupted cell-cell adhesion as reflected by the fragmentation of intercalated discs that connect adjacent cardiac *cells* and the subsequent local misalignment of intracellular actomyosin filaments. At the tissue level, removal of collagen XV led to disorganization of the collagen fibrillar network in the interstitial matrix between cardiomyocytes as well as the accumulation of non-fibrillar fibronectin and fibrin aggregates in this area. Interestingly, the stiffness of *Col15a1*^−/−^ cardiac tissue was significantly increased compared to wildtype. This could be due to the extra-deposition of fibrin aggregates, an ECM protein used to modulate mechanical properties in biomaterials [66]. Another possibility is that collagen XV itself can modulate tissue stiffness. This is highly possible since collagen XV carries charged CS and HS chains that are critical for tissue viscoelasticity [67].

So far, given its location at the outer surface of BMs, collagen XV has been proposed to function as an anchoring protein that links BMs to collagen fibrils of subjacent connective tissues. However, we are far from understanding the whole sequence of events leading to these multiple defects in the myocardium and how these defects relate to one another. Among the open questions are, whether collagen XV is directly involved in the maintenance of cell-cell adhesion integrity, and whether the fragmentation of cardiomyocyte junctions is a mechanical consequence of the cell incapacity to cope up with the increase in tissue stiffness of the overall myocardium.

On an evolutionary perspective, it is interesting to note that a role in heart morphogenesis has also recently been reported for the *Drosophila* Dmp/Mp multiplexin suggesting that the function of multiplexins in the cardiovascular system has been conserved during evolution, although the mechanistic details are quite different. Indeed, collagen XV surrounds the capillary walls and cardiomyocytes in murine heart [50], while in *Drosophila*, the multiplexin is deposited in a polarized way along the heart lumen during the formation of the heart tube and was shown to be necessary and sufficient for the lumen shaping by enhancing Slit/Robo activity [12]. Interestingly, distribution of the invertebrate multiplexin in the heart tube resembles the one of the zebrafish collagen XV-B (Fig. 2A). However, collagen XV function in developing heart has not been investigated in this model yet.

### The peripheral nervous system

Except for one study showing that collagen XV is involved in the regulation of the astrocyte recruitment in developing retina vasculature [68], most of the studies point to a role of collagen XV in the peripheral nervous system. The BM of Schwann cells participate actively to their maturation and to the process of radial sorting leading to myelination of one axon by one Schwann cell [69,70] notably being involved in cell shape changes *via* the activation of the cytoskeleton [71]. Due to the abundance of collagen XV in the BMZ of the peripheral nervous system in mice, Rasi and collaborators [17] analyzed the structure and physiology of the peripheral nerves in *Col15a1*^*−/−*^ mice. They showed that the absence of collagen XV results in altered maturation of peripheral nerves mostly by affecting the process of myelination. Indeed, in the sciatic sensorimotor nerve, lack of collagen XV resulted in abnormal polyaxonal myelination, in the decreased thickness of the myelin sheath as well as in the loosely packed unmyelinated axons of C-fibers [17]. From a physiological point of view, these myelination impairments were essentially reflected by a lower nerve impulse conduction velocity along the sensory nerves, but did not seem to modify the conductivity of motor nerves. Moreover, the authors have shown that double knockout mice in which both *Col15a1* and *Lama4* genes had been inactivated suffer from more severe impaired myelination than the one observed in single knockout mice, suggesting a functional interaction between these two proteins [17]. However, no physical interaction between recombinant laminin α4 and collagen XV has been demonstrated so far. The molecular mechanism through which collagen XV contributes to myelination has not yet been elucidated. The authors suggest that collagen XV may play a structural role in maintaining the integrity between the BM and the surrounding ECM, providing mechanical support for Schwann cells and thus allowing the organization of C-fibers and axon segregation.

In invertebrate models where the collagen XV homologue is highly present in the nervous system, the multiplexin was shown to be particularly involved in axon guidance and migration of neural cells [7,8]. Such function is not surprising as ECM provides neurons with contact-guidance cues necessary for their growth and navigation. Besides the classical axon guidance molecules such as netrin or semaphorin, several ECM components and particularly proteoglycans where shown to be involved in axon pathfinding with their sugar modifications that may also participate to their instructive guidance function [[72], [73], [74]]. In this way, in *C. elegans*, deletion mutagenesis of the collagen XV/XVIII ortholog *cle-1* produced a *cg120* allele mutant allowing the production of a truncated multiplexin deleted from its NC1 domain. In these mutants mechanosensory neurons were mispositioned and motor axons projected on the incorrect side of the ventral cord. Ectopic trimeric NC1 over-expression in the mechanosensory neurons of *cg120* rescued the migration defect phenotype. Interestingly, RNA interference against *cle-1* resulted in similar motor neuron positioning and axon guidance defects with higher penetrance [7]. In Drosophila, the generation of deletion alleles for *dmp*, the drosophila *homologue* of collagen XV/XVIII, also revealed motor axon guidance errors. However, only monomeric endostatin over-expression domain was able to rescue the defects [8]. Interestingly, in zebrafish, the slow muscle precursors, a.k.a. adaxial cells, laid down a collagen XV matrix fingerprint that guides motor axon navigation [18]. Loss (mutant and morphant) and specific gain of *col15a1b* function in slow muscle both provoked pathfinding errors in primary and secondary motor neuron axons [18]. The part of the protein which is responsible for this function has not been determined yet, however it is likely plausible that GAG chains carried by collagen XV-B participate in axon pathfinding as it has been reported for other proteoglycans [74].

## Dysregulation of collagen XV in disease

No inherited human disease has been associated with mutations in the *COL15A1* gene so far. Yet, dysregulation of collagen XV protein expression is a common occurrence in various acquired diseases, from cancer [19] to more specific diseases such as obesity and eye defects [75,76]. However, the molecular mechanisms behind these observations are largely unknown.

### Tumorigenesis

Collagen XV expression and localization have been found to differ in various human cancers when compared with healthy tissue. Together these studies imply a role for collagen XV in tumor microenvironment, affecting *e.g.* the stiffness and integrity of the tumor ECM. Collagen XV has been reported to be lost from the epithelial BM of various invasive tumors, such as ductal breast carcinoma [77], colon adenocarcinoma [78] and skin squamous cell carcinoma [79,80] and instead appears often in the malignant tumor stroma of these cancers. Collagen XV was shown to be expressed in tumoral areas of hepatocellular carcinoma and highly upregulated specifically along the sinusoid-like endothelium implying a role of collagen XV in the vascularization of the tumor [81].

Early studies have focused on the anti-angiogenic properties of restin [31,34,82]. These studies showed that recombinant human restin specifically inhibits the *in vitro* migration of endothelial cells induced by FGF2 and the formation of blood vessels when VEGF is used to induce angiogenesis in CAM (ChorioAllantoic Membrane) angiogenic assays. Human restin inhibited *in vivo* tumor growth when administered systemically in mouse xenografts transplanted with a human renal carcinoma cell line. These early studies tended to associate the anti-tumoral properties of collagen XV with the restin domain. However, several studies have then shown a direct effect of the entire molecule on tumoral cells. Collagen XV was even hypothesized to represent a tumor suppressor acting within the BM which is the first structure being remodelled during the tumor extravasation (reviewed in [83]). Indeed, as mentioned above, collagen XV is lost in the BM of the malignant epithelium in several cancer types like many other BM components [77,78,80], thereby constituting a protective barrier to prevent tumor invasion. Hurskainen and colleagues [33] have also shown that recombinant human collagen XV inhibits the adhesion and migration of fibrosarcoma-derived cells in an adhesion assay using fibronectin as a substrate. In the same line, overexpressing collagen XV in a cervical carcinoma cell line increased the adhesion of these tumor cells to type I collagen and reduced their tumorigenicity *in vivo*. Moreover, Mutolo and collaborators have shown that these two effects were independent of the presence of the restin domain [84]. Clementz and collaborators [19] have studied the mechanism underlying the collagen XV anti-tumoral effects more in-depth in a cellular model of the epithelial–mesenchymal transition (EMT) in pancreatic adenocarcinoma. They have shown that collagen XV overexpressed in these cells interacts with E-cadherin, inhibiting its re-localization, the latter being indispensable for the EMT of tumor cells. Collagen XV interacted also with the DDR1 receptor, thus inhibiting the signaling pathway induced by this receptor that is associated with cell proliferation and migration. Finally, collagen XV has been shown to regulate α2β1 integrin signaling pathway, and it has been hypothesized that the same mechanism could be responsible for the decrease in N-cadherin expression (essential for cell migration) observed in this study. All these data have resulted in a model illustrating the molecular mechanism underlying the tumor suppressor function of collagen XV [83].

### Ischemia

Blood clot of a brain vessel can induce ischemic stroke that is a main cause of death worldwide. To study this disease experimentally, mice are subjected to thromboembolic stroke provoked by a local injection of thrombin into the middle cerebral artery followed by the only treatment used against ischemic stroke, *i.e.* a reperfusion with intravenous administration of recombinant tissue plasminogen activator (rtPA). While such an induced-stroke in wildtype mice did not influence plasma concentration of collagen XV, rtPA administration after ischemia tended to increase collagen XV level in plasma. Analysis of response to ischemic stroke in *Col15a1*^*−/−*^ mice showed that the lack of collagen XV is protective as the lesion volume in the brain was significantly smaller. Thus, collagen XV deficiency has neuroprotective effects that might be attributed to the increase expression of the pro-angiogenic factor, vascular growth endothelial factor A (VEGF-A) [85]. A similar effect of collagen XV deficiency in ischemia was reported in a model of bilateral renal ischemia/reperfusion established in *Col15a1*^*−/−*^ and *Col18a1*^*−/−*^ mice to evaluate the impact of lack of multiplexins on renal function. Zaferani and collaborators [86] reported a reduced tubular damage and a decrease in the recruitment of neutrophils and macrophages in *Col15a1*/*Col18a1* double mutant mice. In the case of collagen XVIII, they demonstrated that the HS chains participate in macrophage migration *in vitro* [86].

### Atherosclerosis

In 2013, a single nucleotide polymorphism within *COL15A1* has been reported to correlate with atherosclerosis in aged individuals [87]. This modification resides in an epigenetically regulated region of the gene leading to a decrease of *COL15A1* expression. *COL15A1* was also found to be upregulated with age in human and mouse atherosclerosis resulting in abnormal protein deposits in the affected blood vessels. In both cases, the regulation of *COL15A1* expression was controlled epigenetically, with hypomethylation leading to an increase in the gene expression. More recently, the same group used a conditional smooth muscle cell (SMC) specific knockout of *Col15a1* to confirm the implication of collagen XV in the development of the disease [20]. Surprisingly, *Col15a1* KO mice that have been fed to develop atherosclerosis, failed to form advanced lesions and, on the contrary, presented lesion size reduction by 78%. *In vivo* RNA-sequencing analyses of SMC isolated from wild-type and *Col15a1* knock-out mice revealed a decrease in inflammatory pathways. Such results remind the one obtained in the renal ischemic/reperfusion model [86].

### Eye disease

Contrarily to its homologue collagen XVIII, the presence of collagen XV in the eye has not been intensively investigated in mammals. Although both collagens were shown to colocalize in many regions of the eye BM, striking differences were observed such as the presence of collagen XV in some retinal capillaries while collagen XVIII was absent [46]. As *COL18A1* mutations resulted in Knobloch syndrome, a human disease characterized by ocular abnormalities [14], it is not surprising that collagen XV dysregulation was associated with several forms of eye disease.

Keratoconus is a common corneal disease characterized by a progressive thinning and protrusion of the central cornea that leads to irregular myopic astigmatism and blurred vision. In affected corneas, collagen XV positive immunoreactivity was observed in subepithelial fibrosis beneath the Bowman's membrane as well as in the corneal stroma but at lower levels [76]. Of note, collagen XV was also found in the fibrous stroma of scarred cornea [76]. In parallel, another group found in patients suffering of late-onset Fuch's corneal dystrophy an increase in collagen XV in the Descemet's membrane, a BM localized at the interface between the corneal stroma and the endothelium. Nevertheless, several other ECM proteins including type III, VII, XVI collagens and the proteoglycans agrin and versican were also found to be dysregulated [88].

Cuticular Drusen subtype of age-related macular degeneration is characterized by drusen formation in the central region of the retina leading to visual impairment. Whole exome sequencing of patients suffering from Cuticular Drusen subtype allowed identifying a rare pathogenic variant in *COL15A1* [89]. Other *COL15A1* variant was also found to be present in young individuals with primary open angle glaucoma. The finding that the parents were not affected led to the hypothesis that the *COL15A1* variant was not causative but rather took part to the age of disease onset [90]. Interestingly, a rare *COL18A1* variant was also observed in other pedigrees in this study with early-onset disease.

### Fibrosis

Collagen XV is dysregulated in several fibrotic diseases. For instance, a strong immunostaining of the multiplexin was observed in fibrotic areas of the kidney from patients with diabetic glomerulosclerosis [49]. In the same way, while healthy liver contains low amount of collagen XV, *Col15a1* was one of the highest increased gene in a mouse model of cholestatic liver disease that is accompanied with liver damage and hepatic fibrosis [91]. Finally, increased collagen XV amount was also observed in advanced stages of fibrosis in human and rat liver tissue. It is produced by a specialized cell type, the portal myofibroblasts that proliferate during advanced fibrosis to promote angiogenesis [92,93].

On the contrary, *COL15A1* was among the few genes to be down-regulated in fibroblasts from patients with Dupuytren's contracture. This most common inherited connective tissue disease is characterized by abnormal proliferation of fibroblasts, contraction of the palmar fascia and fibrotic tissue [94]. This suggests that *COL15A1* is differently regulated in fibrotic tissues in which it may have distinct functions. Moreover, increased expression of collagen XV has been observed in fibrotic areas for example in tumors [77] and in scarred cornea [76].

### Obesity

Collagen XV was shown to be expressed during *in vitro* adipocyte differentiation [95,96]. The multiplexin expression was increased in obese adipose tissue of mice fed with a high fat diet (HFD) used as a model for obesity. Overexpression of collagen XV in HFD mice accelerated lipid deposition by promoting adipogenic markers and reducing lipolytic markers. Collagen XV enhanced adipocyte differentiation and lipid deposition through reducing its DNA methylation and repressing the cAMP/PKA signaling pathway [75].

## Outlook

Collagen XV is a widespread multiplexin across tissues and organisms and it plays a role in a multitude of physiological and pathological contexts (Fig. 2B). Some of its known functions rely on its structural role as a scaffolding matrix organizer or molecular linker. However, collagen XV is a proteoglycan and a wide range of mechanical functions have been proposed for proteoglycans, varying from preventing collagen fibrils from over-stretching in response to mechanical load [97] to modulating global viscoelastic tissue properties [67]. Collagen XV is a proteoglycan that can modulate through its CS and HS chains the relative mechanical properties of the tissues in which it is deposited. Interestingly, collagen XV along with other BM collagens was shown to enable transmission of mechanical signal between cells and whole artery ECM [98]. In addition, the recent studies have also opened new research avenues such as how does collagen XV that is classically seen as a structural organizer affect migration of such a large repertoire of cell types including neural cell, glial cells and immune cells [68,86]. Yet, from all the present literature, it seems that the current mechanistic view on how collagen XV plays its various roles still remains obscure and it is therefore the next challenging puzzle to solve.

## Declaration of competing interest

The authors declare that they have no known competing financial interests or personal relationships that could have appeared to influence the work reported in this paper.

## References

[1] Bella J., Hulmes D.J.S. (2017). Fibrillar collagens. Sub-Cellular Biochemistry.

[2] Fidler A.L., Boudko S.P., Rokas A., Hudson B.G. (2018). The triple helix of collagens - an ancient protein structure that enabled animal multicellularity and tissue evolution. Journal of Cell Science.

[3] Rehn M., Pihlajaniemi T. (1994). α1(XVIII), a collagen chain with frequent interruptions in the collagenous sequence, a distinct tissue distribution, and homology with type XV collagen. Proceedings of the National Academy of Sciences of the United States of America.

[4] Oh S.P., Kamagata Y., Muragaki Y., Timmons S., Ooshima A., Olsen B.R. (1994). Isolation and sequencing of cDNAs for proteins with multiple domains of Gly-Xaa-Yaa repeats identify a distinct family of collagenous proteins. Proceedings of the National Academy of Sciences of the United States of America.

[5] Nauroy P., Hughes S., Naba A., Ruggiero F. (2018). The in-silico zebrafish matrisome: a new tool to study extracellular matrix gene and protein functions. Matrix Biology.

[6] Bretaud S., Nauroy P., Malbouyres M., Ruggiero F. (2019). Fishing for collagen function: about development, regeneration and disease. Seminars in Cell & Developmental Biology.

[7] Ackley B.D., Crew J.R., Elamaa H., Pihlajaniemi T., Kuo C.J., Kramer J.M. (2001). The NC1/endostatin domain of Caenorhabditis elegans type XVIII collagen affects cell migration and axon guidance. The Journal of Cell Biology.

[8] Meyer F., Moussian B. (2009). Drosophila multiplexin (Dmp) modulates motor axon pathfinding accuracy. Development, Growth & Differentiation.

[9] Pagnon-Minot A., Malbouyres M., Haftek-Terreau Z., Kim H.R., Sasaki T., Thisse C., Thisse B., Ingham P.W., Ruggiero F., Le Guellec D., Collagen X.V. (2008). A novel factor in zebrafish notochord differentiation and muscle development. Developmental Biology.

[10] Bretaud S., Pagnon-Minot A., Guillon E., Ruggiero F., Le Guellec D. (2011). Characterization of spatial and temporal expression pattern of Col15a1b during zebrafish development. Gene Expression Patterns.

[11] Momota R., Naito I., Ninomiya Y., Ohtsuka A. (2011). Drosophila type XV/XVIII collagen, Mp, is involved in wingless distribution. Matrix Biology.

[12] Harpaz N., Ordan E., Ocorr K., Bodmer R., Volk T. (2013). Multiplexin promotes heart but not aorta morphogenesis by polarized enhancement of slit/Robo activity at the heart lumen. PLoS Genetics.

[13] Li D., Clark C.C., Myers J.C. (2000). Basement membrane zone type XV collagen is a disulfide-bonded chondroitin sulfate proteoglycan in human tissues and cultured cells. The Journal of Biological Chemistry.

[14] Heljasvaara R., Aikio M., Ruotsalainen H., Pihlajaniemi T. (2017). Collagen XVIII in tissue homeostasis and dysregulation — lessons learned from model organisms and human patients. Matrix Biology.

[15] Eklund L., Piuhola J., Komulainen J., Sormunen R., Ongvarrasopone C., Fassler R., Muona A., Ilves M., Ruskoaho H., Takala T.E.S., Pihlajaniemi T. (2001). Lack of type XV collagen causes a skeletal myopathy and cardiovascular defects in mice. Proceedings of the National Academy of Sciences.

[16] Rasi K., Piuhola J., Czabanka M., Sormunen R., Ilves M., Leskinen H., Rysä J., Kerkelä R., Janmey P., Heljasvaara R., Peuhkurinen K., Vuolteenaho O., Ruskoaho H., Vajkoczy P., Pihlajaniemi T., Eklund L. (2010). Collagen XV is necessary for modeling of the extracellular matrix and its deficiency predisposes to cardiomyopathy. Circulation Research.

[17] Rasi K., Hurskainen M., Kallio M., Staven S., Sormunen R., Heape A.M., Avila R.L., Kirschner D., Muona A., Tolonen U., Tanila H., Huhtala P., Soininen R., Pihlajaniemi T. (2010). Lack of collagen XV impairs peripheral nerve maturation and, when combined with Laminin-411 deficiency, leads to basement membrane abnormalities and sensorimotor dysfunction. The Journal of Neuroscience.

[18] Guillon E., Bretaud S., Ruggiero F. (2016). Slow muscle precursors lay down a collagen XV matrix fingerprint to guide motor axon navigation. The Journal of Neuroscience.

[19] Clementz A.G., Mutolo M.J., Leir S.H., Morris K.J., Kucybala K., Harris H., Harris A. (2013). Collagen XV inhibits epithelial to mesenchymal transition in pancreatic adenocarcinoma cells. PLoS One.

[20] Durgin B.G., Cherepanova O.A., Gomez D., Karaoli T., Alencar G.F., Butcher J.T., Zhou Y.Q., Bendeck M.P., Isakson B.E., Owens G.K., Connelly J.J. (2017). Smooth muscle cell-specific deletion of col15α1 unexpectedly leads to impaired development of advanced atherosclerotic lesions. American Journal of Physiology. Heart and Circulatory Physiology.

[21] Huebner K., Cannizzaro L.A., Jabs E.W., Kiyirikko S., Manzone H., Pihlajaniemi T., Myers J.C. (1992). Chromosomal assignment of a gene encoding a new collagen type (COL15A1) to 9q21 → q22. Genomics.

[22] Hägg P.M., Horelli-Kuitunen N., Eklund L., Palotie A., Pihlajaniemi T. (1997). Cloning of mouse type XV collagen sequences and mapping of the corresponding gene to 4B1-3: comparison of mouse and human α1(XV) collagen sequences indicates divergence in the number of small collagenous domains. Genomics..

[23] Eklund L., Muona A., Liétard J., Pihlajaniemi T. (2000). Structure of the mouse type XV collagen gene, Col15a1, comparison with the human COL15A1 gene and functional analysis of the promoters of both genes. Matrix Biology.

[24] Myers J.C., Kivirikko S., Gordon M.K., Dion A.S., Pihlajaniemi T. (1992). Identification of a previously unknown human collagen chain, α1(XV), characterized by extensive interruptions in the triple-helical region. Proceedings of the National Academy of Sciences of the United States of America.

[25] Kivirikko S., Heinamaki P., Rehn M., Honkanen N., Myers J.C., Pihlajaniemi T. (1994). Primary structure of the α1 chain of human type XV collagen and exon- intron organization in the 3′ region of the corresponding gene. The Journal of Biological Chemistry.

[26] Ricard-Blum S., Ruggiero F. (2005). The collagen superfamily: from the extracellular matrix to the cell membrane. Pathologie et Biologie.

[27] Wirz J.A., Boudko S.P., Lerch T.F., Chapman M.S., Bächinger H.P. (2011). Crystal structure of the human collagen XV trimerization domain: a potent trimerizing unit common to multiplexin collagens. Matrix Biology.

[28] Bogin O., Kvansakul M., Rom E., Singer J., Yayon A., Hohenester E. (2002). Insight into Schmid metaphyseal chondrodysplasia from the crystal structure of the collagen X NC1 domain trimer. Structure..

[29] Sundaramoorthy M., Meiyappan M., Todd P., Hudson B.G. (2002). Crystal structure of NC1 domains: structural basis for type IV collagen assembly in basement membranes. The Journal of Biological Chemistry.

[30] Cuesta Á.M., Sánchez-Martín D., Blanco-Toribio A., Villate M., Enciso-Álvarez K., Alvarez-Cienfuegos A., Sainz-Pastor N., Sanz L., Blanco F.J., Álvarez-Vallina L. (2012). Improved stability of multivalent antibodies containing the human collagen XV trimerization domain. MAbs..

[31] Sasaki T., Larsson H., Tisi D., Claesson-Welsh L., Hohenester E., Timpl R. (2000). Endostatins derived from collagens XV and XVIII differ in structural and binding properties, tissue distribution and anti-angiogenic activity. Journal of Molecular Biology.

[32] Myers J.C., Amenta P.S., Dion A.S., Sciancalepore J.P., Nagaswami C., Weisel J.W., Yurchenco P.D. (2007). The molecular structure of human tissue type XV presents a unique conformation among the collagens. The Biochemical Journal.

[33] Hurskainen M., Ruggiero F., Hägg P., Pihlajaniemi T., Huhtala P. (2010). Recombinant human collagen XV regulates cell adhesion and migration. The Journal of Biological Chemistry.

[34] Ramchandran R., Dhanabal M., Volk R., Waterman M.J.F., Segal M., Lu H., Knebelmann B., Sukhatme V.P. (1999). Antiangiogenic activity of restin, NC10 domain of human collagen XV: comparison to endostatin. Biochemical and Biophysical Research Communications.

[35] Davis G.E., Bayless K.J., Davis M.J., Meininger G.A. (2000). Regulation of tissue injury responses by the exposure of matricryptic sites within extracellular matrix molecules. The American Journal of Pathology.

[36] Sasaki T., Fukai N., Mann K., Göhring W., Olsen B.R., Timpl R. (1998). Structure, function and tissue forms of the C-terminal globular domain of collagen XVIII containing the angiogenesis inhibitor endostatin. The EMBO Journal.

[37] John H., Preissner K.T., Forssmann W.G., Ständker L. (1999). Novel glycosylated forms of human plasma endostatin and circulating endostatin-related fragments of collagen XV. Biochemistry..

[38] Lee J.H., Isayeva T., Larson M.R., Sawant A., Cha H.R., Chanda D., Chesnokov I.N., Ponnazhagan S. (2015). Endostatin: a novel inhibitor of androgen receptor function in prostate cancer. Proceedings of the National Academy of Sciences of the United States of America.

[39] Amenta P.S., Scivoletti N.A., Newman M.D., Sciancalepore J.P., Li D., Myers J.C. (2005). Proteoglycan-collagen XV in human tissues is seen linking banded collagen fibers subjacent to the basement membrane. The Journal of Histochemistry and Cytochemistry.

[40] Saied-Santiago K., Bülow H.E. (2018). Diverse roles for glycosaminoglycans in neural patterning. Developmental Dynamics.

[41] Iozzo R.V., San Antonio J.D. (2001). Heparan sulfate proteoglycans: heavy hitters in the angiogenesis arena. The Journal of Clinical Investigation.

[42] Mikami T., Kitagawa H. (2013). Biosynthesis and function of chondroitin sulfate. Biochimica et Biophysica Acta, General Subjects.

[43] Sarrazin S., Lamanna W.C., Esko J.D. (2011). Heparan sulfate proteoglycans. Cold Spring Harbor Perspectives in Biology.

[44] Culav E.M., Clark C.H., Merrilees M.J. (1999). Connective tissues: matrix composition and its relevance to physical therapy. Physical Therapy.

[45] Halfter W., Dong S., Schurer B., Cole G.J. (1998). Collagen XVIII is a basement membrane heparan sulfate proteoglycan. The Journal of Biological Chemistry.

[46] Ylikärppä R., Eklund L., Sormunen R., Muona A., Fukai N., Olsen B.R., Pihlajaniemi T. (2003). Double knockout mice reveal a lack of major functional compensation between collagens XV and XVIII. Matrix Biology.

[47] Kivirikko S., Saarela J., Myers J.C., Autio-Harmainen H., Pihlajaniemi T. (1995). Distribution of type XV collagen transcripts in human tissue and their production by muscle cells and fibroblasts. The American Journal of Pathology.

[48] Myers J.C., Dion A.S., Abraham V., Amenta P.S. (1996). Type XV collagen exhibits a widespread distribution in human tissues but a distinct localization in basement membrane zones. Cell and Tissue Research.

[49] Hägg P.M., Hägg P.O., Peltonen S., Autio-Harmainen H., Pihlajaniemi T. (1997). Location of type XV collagen in human tissues and its accumulation in the interstitial matrix of the fibrotic kidney. The American Journal of Pathology.

[50] Muona A., Eklund L., Väisänen T., Pihlajaniemi T. (2002). Developmentally regulated expression of type XV collagen correlates with abnormalities in Col15a1−/− mice. Matrix Biology.

[51] Tomono Y., Naito I., Ando K., Yonezawa T., Sado Y., Hirakawa S., Arata J., Okigaki T., Ninomiya Y. (2002). Epitope-defined monoclonal antibodies against multiplexin collagens demonstrate that type XV and XVIII collagens are expressed in specialized basement membranes. Cell Structure and Function.

[52] Ishii M., Koike C., Igarashi A., Yamanaka K., Pan H., Higashi Y., Kawaguchi H., Sugiyama M., Kamata N., Iwata T., Matsubara T., Nakamura K., Kurihara H., Tsuji K., Kato Y. (2005). Molecular markers distinguish bone marrow mesenchymal stem cells from fibroblasts. Biochemical and Biophysical Research Communications.

[53] Lisignoli G., Codeluppi K., Todoerti K., Manferdini C., Piacentini A., Zini N., Grassi F., Cattini L., Piva R., Rizzoli V., Facchini A., Giuliani N., Neri A. (2009). Gene array profile identifies collagen type XV as a novel human osteoblast-secreted matrix protein. Journal of Cellular Physiology.

[54] Manferdini C., Zini N., Gabusi E., Paolella F., Lambertini E., Penolazzi L., Piva R., Lisignoli G. (2018). Immunoelectron microscopic localization of Collagen type XV during human mesenchymal stem cells mineralization. Connective Tissue Research.

[55] Gabusi E., Manferdini C., Grassi F., Piacentini A., Cattini L., Filardo G., Lambertini E., Piva R., Zini N., Facchini A., Lisignoli G. (2012). Extracellular calcium chronically induced human osteoblasts effects: specific modulation of osteocalcin and collagen type XV. Journal of Cellular Physiology.

[56] Lisignoli G., Lambertini E., Manferdini C., Gabusi E., Penolazzi L., Paolella F., Angelozzi M., Casagranda V., Piva R. (2017). Collagen type XV and the “osteogenic status”. Journal of Cellular and Molecular Medicine.

[57] Singh A., Yadav C.B., Tabassum N., Bajpeyee A.K., Verma V. (2019). Stem cell niche: dynamic neighbor of stem cells. European Journal of Cell Biology.

[58] Izzi V., Heljasvaara R., Heikkinen A., Karppinen S.-M., Koivunen J., Pihlajaniemi T. (2019). Exploring the roles of MACIT and multiplexin collagens in stem cells and cancer. Seminars in Cancer Biology.

[59] Pujic Z., Omori Y., Tsujikawa M., Thisse B., Thisse C., Malicki J. (2006). Reverse genetic analysis of neurogenesis in the zebrafish retina. Developmental Biology.

[60] Recher G., Jouralet J., Brombin A., Heuze A., Mugniery E., Hermel J.-M., Desnoulez S., Savy T., Herbomel P., Bourrat F., Peyrieras N., Jamen F., Joly J.-S. (2013). Zebrafish midbrain slow-amplifying progenitors exhibit high levels of transcripts for nucleotide and ribosome biogenesis. Development..

[61] Albadri S., Naso F., Thauvin M., Gauron C., Parolin C., Duroure K., Vougny J., Fiori J., Boga C., Vriz S., Calonghi N., Del Bene F. (2019). Redox signaling via lipid peroxidation regulates retinal progenitor cell differentiation. Developmental Cell.

[62] Lambertini E., Penolazzi L., Angelozzi M., Bergamin L.S., Manferdini C., Vieceli Dalla F., Sega F., Paolella G., Lisignoli R.P. (2018). Hypoxia preconditioning of human MSCs: a direct evidence of HIF-1α and collagen type XV correlation. Cellular Physiology and Biochemistry.

[63] Dietrich-Ntoukas T., Hofmann-Rummelt C., Kruse F.E., Schlötzer-Schrehardt U. (2012). Comparative analysis of the basement membrane composition of the human limbus epithelium and amniotic membrane epithelium. Cornea..

[64] Rygh C.B., Løkka G., Heljasvaara R., Taxt T., Pavlin T., Sormunen R., Pihlajaniemi T., Curry F.R., Tenstad O., Reed R.K. (2014). Image-based assessment of microvascular function and structure in collagen XV- and XVIII-deficient mice. The Journal of Physiology.

[65] Momota R., Narasaki M., Komiyama T., Naito I., Ninomiya Y., Ohtsuka A. (2013). Drosophila type XV/XVIII collagen mutants manifest integrin mediated mitochondrial dysfunction, which is improved by cyclosporin A and losartan. The International Journal of Biochemistry & Cell Biology.

[66] Duong H., Wu B., Tawil B. (2009). Modulation of 3D fibrin matrix stiffness by intrinsic fibrinogen-thrombin compositions and by extrinsic cellular activity. Tissue Engineering Part A.

[67] Lujan T.J., Underwood C.J., Jacobs N.T., Weiss J.A. (2009). Contribution of glycosaminoglycans to viscoelastic tensile behavior of human ligament. Journal of Applied Physiology.

[68] Hurskainen M., Eklund L., Hägg P.O., Fruttiger M., Sormunen R., Ilves M., Pihlajaniemi T. (2005). Abnormal maturation of the retinal vasculature in type XVIII collagen/endostatin deficient mice and changes in retinal glial cells due to lack of collagen types XV and XVIII. The FASEB Journal.

[69] Bunge R. (1986). Linkage between axonal ensheathment and basal lamina production by Schwann cells. Annual Review of Neuroscience.

[70] McKee K.K., Yang D.H., Patel R., Chen Z.L., Strickland S., Takagi J., Sekiguchi K., Yurchenco P.D. (2012). Schwann cell myelination requires integration of laminin activities. Journal of Cell Science.

[71] Fernandez-Valle C., Gorman D., Gomez A.M., Bunge M.B. (1997). Actin plays a role in both changes in cell shape and gene-expression associated with Schwann cell myelination. The Journal of Neuroscience.

[72] Masu M. (2016). Proteoglycans and axon guidance: a new relationship between old partners. Journal of Neurochemistry.

[73] Yu P., Pearson C.S., Geller H.M. (2018). Flexible roles for proteoglycan sulfation and receptor signaling. Trends in Neurosciences.

[74] Holt C.E., Dickson B.J. (2005). Sugar codes for axons?. Neuron..

[75] Liu G., Li M., Xu Y., Wu S., Saeed M., Sun C. (2017). ColXV promotes adipocyte differentiation via inhibiting DNA methylation and cAMP/PKA pathway in mice. Oncotarget..

[76] Määttä M., Heljasvaara R., Sormunen R., Pihlajaniemi T., Autio-Harmainen H., Tervo T. (2006). Differential expression of collagen types XVIII/endostatin and XV in normal, keratoconus, and scarred human corneas. Cornea..

[77] Amenta P.S., Hadad S., Lee M.T., Barnard N., Li D., Myers J.C. (2003). Loss of types XV and XIX collagen precedes basement membrane invasion in ductal carcinoma of the female breast. The Journal of Pathology.

[78] Amenta P.S., Briggs K., Xu K., Gamboa E., Jukkola A.F., Li D., Myers J.C. (2000). Type XV collagen in human colonic adenocarcinomas has a different distribution than other basement membrane zone proteins. Human Pathology.

[79] Fukushige T., Kanekura T., Ohuchi E., Shinya T., Kanzaki T. (2005). Immunohistochemical studies comparing the localization of type XV collagen in normal human skin and skin tumors with that of type IV collagen. The Journal of Dermatology.

[80] Karppinen S.M., Honkanen H.K., Heljasvaara R., Riihilä P., Autio-Harmainen H., Sormunen R., Harjunen V., Väisänen M.R., Väisänen T., Hurskainen T., Tasanen K., Kähäri V.M., Pihlajaniemi T. (2016). Collagens XV and XVIII show different expression and localisation in cutaneous squamous cell carcinoma: type XV appears in tumor stroma, while XVIII becomes upregulated in tumor cells and lost from microvessels. Experimental Dermatology.

[81] Kimura K., Nakayama M., Naito I., Komiyama T., Ichimura K., Asano H., Tsukuda K., Ohtsuka A., Oohashi T., Miyoshi S., Ninomiya Y. (2016). Human collagen XV is a prominent histopathological component of sinusoidal capillarization in hepatocellular carcinogenesis. International Journal of Clinical Oncology.

[82] Xu R., Xin L., Fan Y., Meng H.-R., Li Z.-P., Gan R.-B. (2002). Mouse restin inhibits bovine aortic endothelial cell proliferation and causes cell apoptosis. Sheng Wu Hua Xue Yu Sheng Wu Wu Li Xue Bao (Shanghai).

[83] Clementz A.G., Harris A. (2013). Collagen XV: exploring its structure and role within the tumor microenvironment. Molecular Cancer Research.

[84] Mutolo M.J., Morris K.J., Leir S., Caffrey T.C., Lewandowska M.A., Hollingsworth M.A., Harris A. (2012). Tumor suppression by collagen XV is independent of the restin domain. Matrix Biology.

[85] Dhungana H., Huuskonen M.T., Pihlajaniemi T., Heljasvaara R., Vivien D., Kanninen K.M., Malm T., Koistinaho J., Lemarchant S. (2017). Lack of collagen XV is protective after ischemic stroke in mice. Cell Death & Disease.

[86] Zaferani A., Talsma D.T., Yazdani S., Celie J.W.A.M., Aikio M., Heljasvaara R., Navis G.J., Pihlajaniemi T., Van Den Born J. (2014). Basement membrane zone collagens XV and XVIII/proteoglycans mediate leukocyte influx in renal ischemia/reperfusion. PLoS One.

[87] Connelly J.J., Cherepanova O.A., Doss J.F., Karaoli T., Lillard T.S., Markunas C.A., Nelson S., Wang T., Ellis P.D., Langford C.F., Haynes C., Seo D.M., Goldschmidt-Clermont P.J., Shah S.H., Kraus W.E., Hauser E.R., Gregory S.G. (2013). Epigenetic regulation of COL15A1 in smooth muscle cell replicative aging and atherosclerosis. Human Molecular Genetics.

[88] Weller J.M., Zenkel M., Schlötzer-Schrehardt U., Bachmann B.O., Tourtas T., Kruse F.E. (2014). Extracellular matrix alterations in late-onset Fuchs’ corneal dystrophy. Investigative Ophthalmology and Visual Science.

[89] Duvvari M.R., Van De Ven J.P.H., Geerlings M.J., Saksens N.T.M., Bakker B., Henkes A., Neveling K., Del Rosario M., Westra D., Van Den Heuvel L.P.W.J., Schick T., Fauser S., Boon C.J.F., Hoyng C.B., De Jong E.K., Den Hollander A.I. (2016). Whole exome sequencing in patients with the cuticular drusen subtype of age-related macular degeneration. PLoS One.

[90] Wiggs J.L., Howell G.R., Linkroum K., Abdrabou W., Hodges E., Braine C.E., Pasquale L.R., Hannon G.J., Haines J.L., John S.W.M. (2013). Variations in COL15A1 and COL18A1 influence age of onset of primary open angle glaucoma. Clinical Genetics.

[91] Sun J., Wang J., Zhang N., Yang R., Chen K., Kong D. (2019). Identification of global mRNA expression profiles and comprehensive bioinformatic analyses of abnormally expressed genes in cholestatic liver disease. Gene..

[92] Lemoinne S., Cadoret A., Rautou P.E., El Mourabit H., Ratziu V., Corpechot C., Rey C., Bosselut N., Barbu V., Wendum D., Feldmann G., Boulanger C., Henegar C., Housset C., Thabut D. (2015). Portal myofibroblasts promote vascular remodeling underlying cirrhosis formation through the release of microparticles. Hepatology..

[93] Lemoinne S., Thabut D., Housset C. (2016). Portal myofibroblasts connect angiogenesis and fibrosis in liver. Cell and Tissue Research.

[94] Satish L., LaFramboise W.A., O’Gorman D.B., Johnson S., Janto B., Gan B.S., Baratz M.E., Hu F.Z., Post J.C., Ehrlich G.D., Kathju S. (2008). Identification of differentially expressed genes in fibroblasts derived from patients with Dupuytren’s contracture. BMC Medical Genetics.

[95] Molina H., Yang Y., Ruch T., Kim J.W., Mortensen P., Otto T., Nalli A., Tang Q.Q., Daniel Lane M., Chaerkady R., Pandey A. (2009). Temporal profiling of the adipocyte proteome during differentiation using a five-plex SILAC based strategy. Journal of Proteome Research.

[96] Mori S., Kiuchi S., Ouchi A., Hase T., Murase T. (2014). Characteristic expression of extracellular matrix in subcutaneous adipose tissue development and adipogenesis; comparison with visceral adipose tissue. International Journal of Biological Sciences.

[97] Linka K., Khiêm V.N., Itskov M. (2016). Multi-scale modeling of soft fibrous tissues based on proteoglycan mechanics. Journal of Biomechanics.

[98] Osidak M.S., Osidak E.O., Akhmanova M.A., Domogatsky S.P., Domogatskaya A.S. (2015). Fibrillar, fibril-associated and basement membrane collagens of the arterial wall: architecture, elasticity and remodeling under stress. Current Pharmaceutical Design.

[99] Schlötzer-Schrehardt U., Dietrich T., Saito K., Sorokin L., Sasaki T., Paulsson M., Kruse F.E. (2007). Characterization of extracellular matrix components in the limbal epithelial stem cell compartment. Experimental Eye Research.

[100] Saika S., Okada Y., Miyamoto T., Yamanaka O., Ohnishi Y., Yamanaka A., Ooshima A. (2004). Protein expression pattern of collagen type XV in mouse cornea. Graefe’s Archive for Clinical and Experimental Ophthalmology.

[101] Muragaki Y., Abe N., Ninomiya Y., Olsen B.R., Ooshima A. (1994). The human alpha 1(XV) collagen chain contains a large amino-terminal non-triple helical domain with a tandem repeat structure and homology to alpha 1(XVIII) collagen. The Journal of Biological Chemistry.

[102] Landis B. (2017). exome sequencing identifies cadidates genetic modifiers of syndromic and familial thoracic aortic aneurysm severity. Journal of Cardiovascular Translational Research.

